# Sex Differences in CGRP Regulation and Function in the Amygdala in a Rat Model of Neuropathic Pain

**DOI:** 10.3389/fnmol.2022.928587

**Published:** 2022-06-03

**Authors:** Peyton Presto, Volker Neugebauer

**Affiliations:** ^1^Department of Pharmacology and Neuroscience, Texas Tech University Health Sciences Center, Lubbock, TX, United States; ^2^Center of Excellence for Translational Neuroscience and Therapeutics, Texas Tech University Health Sciences Center, Lubbock, TX, United States; ^3^Garrison Institute on Aging, Texas Tech University Health Sciences Center, Lubbock, TX, United States

**Keywords:** amygdala, CGRP, sex differences, plasticity, behavior, neuropathic pain (NP)

## Abstract

The amygdala has emerged as a key player in the emotional response to pain and pain modulation. The lateral and capsular regions of the central nucleus of the amygdala (CeA) represent the “nociceptive amygdala” due to their high content of neurons that process pain-related information. These CeA divisions are the targets of the spino-parabrachio-amygdaloid pain pathway, which is the predominant source of calcitonin gene-related peptide (CGRP) within the amygdala. Changes in lateral and capsular CeA neurons have previously been observed in pain models, and synaptic plasticity in these areas has been linked to pain-related behavior. CGRP has been demonstrated to play an important role in peripheral and spinal mechanisms, and in pain-related amygdala plasticity in male rats in an acute arthritis pain model. However, the role of CGRP in chronic neuropathic pain-related amygdala function and behaviors remains to be determined for both male and female rats. Here we tested the hypothesis that the CGRP1 receptor is involved in neuropathic pain-related amygdala activity, and that blockade of this receptor can inhibit neuropathic pain behaviors in both sexes. CGRP mRNA expression levels in the CeA of male rats were upregulated at the acute stage of the spinal nerve ligation (SNL) model of neuropathic pain, whereas female rats had significantly higher CGRP and CGRP receptor component expression at the chronic stage. A CGRP1 receptor antagonist (CGRP 8-37) administered into the CeA in chronic neuropathic rats reduced mechanical hypersensitivity (von Frey and paw compression tests) in both sexes but showed female-predominant effects on emotional-affective responses (ultrasonic vocalizations) and anxiety-like behaviors (open field test). CGRP 8-37 inhibited the activity of CeA output neurons assessed with calcium imaging in brain slices from chronic neuropathic pain rats. Together, these findings may suggest that CGRP1 receptors in the CeA are involved in neuropathic pain-related amygdala activity and contribute to sensory aspects in both sexes but to emotional-affective pain responses predominantly in females. The sexually dimorphic function of CGRP in the amygdala would make CGRP1 receptors a potential therapeutic target for neuropathic pain relief, particularly in females in chronic pain conditions.

## Introduction

Interactions between sensory, emotional-affective, and cognitive dimensions comprise the highly complex, intense pain experience. The intricate interplay of each of these pain components presents a challenge to identifying effective therapeutic strategies for chronic pain relief, as many therapeutic options are associated with variable efficacy and undesirable side effects (Attal, 2019; Bates et al., 2019). One barrier to the discovery of effective treatment options is the lack of full understanding of mechanisms and targets that are involved in a chronic pain state, particularly with regard to potential sex differences. It is estimated that chronic pain impacts 20% of the global population each year (Breivik et al., 2013; Fayaz et al., 2016; Dahlhamer et al., 2018), with females greatly outnumbering males as chronic pain patients (Ruau et al., 2012). However, many currently available analgesics have limited effectiveness in female patients due to the targeting of male-specific pain processing mechanisms (Mogil, 2020; Shansky and Murphy, 2021). Therefore, there is an urgent need to identify sex-specific targets that can lead to the development of improved therapeutic options for pain treatment in male and female patients.

The amygdala is a limbic brain structure that has been revealed to play a critical role in the emotional-affective dimension of pain and pain modulation (Veinante et al., 2013; Neugebauer, 2015, 2020; Neugebauer et al., 2020). The amygdala attaches emotional significance to sensory information from various pain states (Neugebauer et al., 2004; Thompson and Neugebauer, 2017) and connects to descending pain modulatory system structures and other nervous system structures involved in behavioral, emotional, and cognitive functions (Becker and Carrasquillo, 2019; Hua et al., 2020; Liu et al., 2021; Weera et al., 2021). The amygdala is comprised of functionally and anatomically distinct nuclei. The central nucleus of the amygdala (CeA) serves as the primary amygdala output center, and its lateral and capsular divisions receive nociceptive information through the spino-parabrachio-amygdaloid pain pathway (Gauriau and Bernard, 2002; Thompson and Neugebauer, 2017; Kato et al., 2018; Neugebauer, 2020). As neurons in the lateral and capsular regions predominately respond to noxious stimuli (Bernard et al., 1992; Neugebauer and Weidong, 2002), these divisions are often said to comprise the “nociceptive amygdala” (Neugebauer et al., 2004).

Calcitonin gene-related peptide (CGRP) is a 37 amino acid peptide that binds to the G-protein-coupled CGRP1 receptor to activate adenylyl cyclase and protein kinase A (PKA; Russo, 2015). The CGRP1 receptor is composed of three predominant subunits: the seven-transmembrane calcitonin-like receptor (CLR) protein; a receptor activity modifying protein (Ramp1), which is critical for the specificity of CGRP binding and cell surface receptor expression; and the CGRP receptor component protein (RCP), which facilitates coupling to cAMP signaling (Poyner et al., 2002; Barwell et al., 2013; Dickerson, 2013; Russo, 2015; Iyengar et al., 2017; Neugebauer et al., 2020). In the amygdala, CGRP serves as an important molecular marker of the lateral and capsular regions of the CeA due to the CGRP-immunoreactive terminals of fibers from the lateral parabrachial nucleus that synapse in these areas (Kruger et al., 1988; Shimada et al., 1989; Dobolyi et al., 2005; D’Hanis et al., 2007; Palmiter, 2018). CGRP modulates synaptic input from the parabrachial nucleus to the CeA (Han et al., 2005, 2015; Okutsu et al., 2017; Shinohara et al., 2017) and plays a critical role in synaptic plasticity in the CeA in an arthritis pain model (Han et al., 2005). CeA cell types targeted by CGRP input from the parabrachial area include neurons expressing protein kinase C delta or corticotropin releasing factor (CRF; Neugebauer et al., 2020). We have previously shown that the administration of a selective CGRP1 receptor antagonist (CGRP 8-37) into the CeA can inhibit pain behaviors in an acute arthritis model (Han et al., 2005), suggesting that CGRP-mediated mechanisms may influence pain-related amygdala function. However, the role of CGRP in CeA pain mechanisms and sex differences in a chronic neuropathic pain state remains to be determined.

The purpose of this study was to examine the role of CGRP in neuropathic pain-related CeA function and behaviors with regard to sex and stage of neuropathic pain development. We characterized mRNA expression profiles of CGRP and CGRP1 receptor components in adult male and female rats at the acute and chronic phases of the rat spinal nerve ligation (SNL) model of neuropathic pain. We also subjected adult male and female neuropathic rats to behavioral tests before and after the blockade of the CGRP1 receptor in the CeA, and we examined the effects of this blockade on CeA-CRF neuronal cell activity. Our previous work implicated CeA-CRF neurons in pain modulation (Mazzitelli et al., 2021, 2022). Our findings point to a previously unexplored, sexually dimorphic role of CGRP in neuropathic pain-related processing within the CeA.

## Materials and Methods

### Animals

Adult male and female Sprague-Dawley rats (150–300 g, 12 weeks of age at time of testing) were group-housed in a temperature-controlled room under a 12 h day/night cycle with unrestricted access to food and water. On each experimental day, rats were transferred from the animal facility and allowed to acclimate to the laboratory for at least 1 h prior to testing. All experimental procedures were approved by the Institutional Animal Care and Use Committee (IACUC, protocol #14006) of Texas Tech University Health Sciences Center (TTUHSC) and conformed to the guidelines of the International Association for the Study of Pain (IASP) and of the National Institutes of Health (NIH).

### Experimental Protocol

mRNA expression levels of CGRP and two of the CGRP1 receptor components, CLR and Ramp1, were measured (see Section “qRT-PCR”) in the right CeA of rats in the acute and chronic phases of neuropathic pain (see Section “Neuropathic Pain Model”). The effects of the selective CGRP1 receptor peptide antagonist (CGRP 8-37) compared to artificial cerebrospinal fluid (ACSF) vehicle control were tested in chronic neuropathic rats in behavioral (see Section “Behaviors”) and multiphoton calcium imaging (see Section “Calcium Imaging”) experiments. For behavioral experiments, a guide cannula was implanted (see Section “Drug Application in Awake Animals”) 1 week before stereotaxic drug application into the CeA by microdialysis. Behavioral assays were performed at 20 min during continued drug (or vehicle) administration. For calcium imaging experiments, CGRP 8-37 or ACSF was applied directly to the brain slice *via* gravity-driven superfusion. The right CeA was targeted in all molecular, behavioral, and calcium imaging experiments, as evidence suggests right-hemispheric lateralization of pain processing and modulation (Carrasquillo and Gereau IV, 2008; Ji and Neugebauer, 2009; Gonçalves and Dickenson, 2012; Simons et al., 2014; Nation et al., 2018; Phelps et al., 2019; Allen et al., 2021).

### Neuropathic Pain Model

The well-established spinal nerve ligation (SNL) model of neuropathic pain (Ho Kim and Mo Chung, 1992) was used to create a stable and long-lasting peripheral neuropathy. Rats were anesthetized with isoflurane (2%–3%; precision vaporizer, Harvard Apparatus, Holliston, MA, USA) and underwent surgery in which the left L5 spinal nerve was exposed and tightly ligated using 6–0 sterile silk. In the sham-operated control group, the spinal nerve was exposed but not ligated.

### qRT-PCR

At either the acute phase (1 week post-SNL) or chronic phase (4 weeks post-SNL) of neuropathic pain (see Section “Neuropathic Pain Model”), rats were euthanized by decapitation. Brains were rapidly extracted and oxygenated in ice-cold sucrose-based physiological solution (87 NaCl, 75 sucrose, 25 glucose, 5 KCl, 21 MgCl_2_, 0.5 CaCl_2_, and 1.25 NaH_2_PO_4_). Coronal brain slices (1,000 μm) containing the CeA were prepared using a Vibratome (VT1200S, Leica Biosystems, Nussloch, Germany) as described previously (Thompson et al., 2018; Navratilova et al., 2019; Hein et al., 2021; Mazzitelli et al., 2021). The right CeA was dissected from freshly harvested slices for mRNA analysis. RNA was extracted using the MagMAX-96 Total RNA Isolation Kit (Life Technologies, Carlsbad, CA, USA) and quantified on a NanoDrop 8000 spectrophotometer (Thermo Fisher Scientific, Rockford, IL, USA). Total RNA was reverse transcribed using the High-Capacity cDNA Reverse Transcription Kit with RNase Inhibitor (Thermo Fisher Scientific), and then Taqman Fast Advanced Master Mix (Thermo Fisher Scientific) was used to perform quantitative reverse transcription polymerase chain reactions (qRT-PCR). Applied Biosystems Taqman Gene Expression Assays included (CGRP; *Calca*; Rn01511353_g1), CLR (*Calcrl*; Rn00562334_m1), Ramp1 (*Ramp1*; Rn01427056_m1), β-actin (*Actb*; Rn00667869_m1), Rpl3 (*Rpl3*; Rn01505100_g1), and Rpl29 (*Rpl29*; Rn00820801_g1). Reactions containing 5 ng of cDNA were performed in triplicate using the CFX384 Real-Time System (BioRad, Hercules, CA, USA). Relative expression was determined using the 2^−ΔΔCt^ method with samples normalized to the geometric mean of β-actin, Rpl3, and Rpl29, as this normalization strategy utilized the three genes with the most stable expression in a rat neuropathic pain model (Wan et al., 2010).

### Drug Application in Awake Animals

Stereotaxic drug administration into the CeA by microdialysis was performed 4 weeks after SNL surgery as described before (Kiritoshi et al., 2016; Kim et al., 2017; Thompson et al., 2018; Mazzitelli and Neugebauer, 2019; Hein et al., 2021). Rats were anesthetized with isoflurane (2%–3%; precision vaporizer, Harvard Apparatus) and a small unilateral craniotomy was performed. Using a stereotaxic apparatus (David Kopf Instruments, Tujunga, CA, USA), a guide cannula (CMA/Microdialysis, Solna, Sweden) was inserted into the CeA using the following coordinates (Paxinos and Watson, 1998): 2.5 mm caudal to the bregma, 4.0 mm lateral to the midline, and 7.5 mm deep. The cannula was fixed to the skull using dental acrylic (Plastic One, Roanoke, VA, USA), and local anesthetic (Lidocaine) and antibiotic ointment (Bacitracin) were applied to reduce inflammation and prevent infection. Rats were allowed to recover from cannula implantation for 1 week prior to experimental testing. On the day of the experiment, a microdialysis probe (CMA/Microdialysis 12) protruding 1 mm from the guide cannula was inserted and connected to an infusion pump (Harvard Apparatus) with polyethylene tubing. A selective CGRP1 receptor antagonist (CGRP 8-37; TOCRIS, Minneapolis, MN, USA) was diluted in ACSF (in mM: 117 NaCl, 4.7 KCl, 1.2 NaH_2_PO_4_, 2.5 CaCl_2_, 1.2 MgCl_2_, 25 NaHCO_3_, and 11 glucose) to the final concentration (100 μM), which is 100-fold greater than the target concentration in the tissue to account for the concentration gradient across the dialysis membrane and diffusion in brain tissue. CGRP 8-37 (100 μM) or ACSF was administered at 5 μl/min for at least 20 min prior to behavioral testing to establish equilibrium in the tissue.

### Behaviors

#### Mechanosensitivity

Mechanical withdrawal thresholds were measured using a plantar electronic von Frey anesthesiometer (IITC Life Sciences, Woodland Hills, CA, USA) with the tip applied perpendicularly to the base of the 3rd toe of the left hind paw as described before (Mazzitelli et al., 2022). The tip was applied with increasing force until a flexion reflex was provoked, which was automatically recorded as the paw withdrawal threshold (in grams). Three measurements were recorded and averaged. As the least invasive test, this was always performed prior to any other behavioral assay. Mechanosensitivity was also measured using a paw compression test on the affected hindlimb in the recording system that was also used for vocalization measurements. For that, rats were briefly anesthetized with isoflurane (2%–3%; precision vaporizer, Harvard Apparatus) and placed slightly restrained in a customized holding chamber that allowed hindlimb access (U.S. Patent 7,213,538). Hindlimb withdrawal thresholds were measured after 30 min of habituation to the holding chamber by using a calibrated forceps with a force transducer (see Section “Emotional-Affective Responses”) to compress the left hindpaw with gradually increasing intensity until a reflex response was evoked, as described in our previous studies (Hein et al., 2021; Presto et al., 2021). The withdrawal threshold, defined as the force required to evoke a reflex response, was calculated using the average value from three trials. The combination of mechanosensitivity tests was used to address both the static (von Frey test; mediated by sensitized peripheral nociceptors) and dynamic (paw compression test; mediated by primary afferent inputs from deep tissue into the central nervous system) components of mechanical hyperalgesia (Koltzenburg et al., 1992; Ochoa and Yarnitsky, 1993; La and Chung, 2017).

#### Emotional-Affective Responses

Emotional-affective responses were assessed by measuring vocalizations in the ultrasonic (25 ± 4 kHz) range, as described in our previous studies (Ji et al., 2018; Mazzitelli and Neugebauer, 2019; Hein et al., 2021; Mazzitelli et al., 2021; Presto et al., 2021). Rats were briefly anesthetized with isoflurane (2%–3%; precision vaporizer, Harvard Apparatus) and placed in the custom-designed recording chamber for stable recordings of vocalizations evoked by noxious stimulation. After the rat recovered from anesthesia and was habituated to the recording chamber for 30 min, the hindlimb withdrawal thresholds were evaluated in the paw pressure test (see Section “Mechanosensitivity”) before the calibrated forceps with a force transducer was used for vocalization assays. Vocalizations were evoked by a brief (10 s) noxious (500 g/6 mm^2^) stimulus applied to the left hind paw. These vocalizations were automatically detected for 1 min using a full-spectrum USB microphone (max sampling rate: 384 kHz), and the ultrasonic component of the vocalizations following the onset of the mechanical stimulus was analyzed using UltraVox 3.2 software (Noldus Information Technology, Leesburg, VA, USA). At the conclusion of each experiment, the durations (in ms) of each individual ultrasonic call were summed for each 1-min recording period to give the total duration of ultrasonic vocalizations for each rat.

#### Anxiety-Like Behavior

The open field test (OFT) was used to measure exploratory behavior in the peripheral and central zones of an open arena (70 cm × 70 cm) with acrylic walls (height, 45 cm). Rat movements were recorded for 15 min using a computerized video tracking and analysis system (EthoVision XT 11 software, Noldus Information Technology) as described previously (Ji et al., 2018; Hein et al., 2021; Presto et al., 2021). Time spent in the center of the arena was calculated during the first 5 min of each experimental trial. Avoidance of the center of the arena is interpreted as anxiety-like behavior (Prut and Belzung, 2003; Seibenhener and Wooten, 2015).

### Calcium Imaging

Rats were anesthetized with isoflurane (2%–3%; precision vaporizer, Harvard Apparatus) and a stereotaxic frame (David Kopf Instruments) was used to inject AAV5-Syn-Flex-GCaMP7 s into the right CeA 5–6 weeks before brain slices were obtained to allow viral vector-mediated expression of a fluorescent calcium sensor. SNL surgery was performed 4 weeks before brain slices were obtained (chronic neuropathic pain stage). On the day of the experiment, coronal brain slices (400 μm) were quickly removed and immersed in oxygenated ACSF at 35°C for at least 1 h before being transferred to the recording chamber and superfused with ACSF (~2 ml/min) as described previously (Hein et al., 2021). One or two brain slices per animal were used. A multiphoton system (Intelligent Imaging Innovations, Inc., 3i, Denver, CO, USA) equipped with a VIVO^TM^2-Photon 200 Microscopy Workstation was utilized to record calcium transients evoked in CeA-CRF neurons by electrical stimulation (1.5 mA, 0.6 ms) of the dorsomedial fiber tract containing presumed axons from the parabrachial area (Neugebauer et al., 2003; Ikeda et al., 2007). CeA-CRF neurons receive CGRP input from the parabrachial nucleus (Neugebauer et al., 2020) and modulate pain behaviors (Mazzitelli et al., 2021, 2022). The 2-photon excitation light was generated by a mode-locked Ti: Sapphire laser (920 nm; model Mai-Tai DeepSee, Spectra-Physics, Santa Clara, CA, USA). A 20×/1.0 NA water-immersion lens (Zeiss, Germany) was used for neuronal cluster imaging. Images were acquired at a rate of 0.85 frames/s with a resolution of 512 × 512 pixels, which corresponded to an image plane of 350 × 350 μm. The CeA-CRF neurons that responded to electrical stimulus were selected for further analysis. Fluorescent activity and background (ΔF/F_0_) were analyzed using SlideBook 6 (3i, Denver, CO, USA). CeA-CRF neuronal responses were recorded 10 min before and 20 min during CGRP 8-37 application (1 μM), followed by washout with ACSF for 40 min. Neuronal responses could also be blocked by the application of an AMPA/kainate receptor antagonist (6-cyano-7-nitroquinoxaline-2,3-dione, CNQX; 20 μM dissolved in ACSF), consistent with a glutamatergic synaptic drive onto CeA-CRF neurons.

### Statistical Analysis

Statistical significance was accepted at the level *P* < 0.05. All averaged values are presented as means ± SEM. GraphPad Prism 9.0 software (Graph-Pad Software, San Diego, CA, USA) was used for all statistical analyses. For qRT-PCR experiments, two-way ANOVA with Tukey *post-hoc* tests were used for multiple comparisons since sample sizes for each group were equal. For behavioral experiments, two-way ANOVA (repeated measures if appropriate) with Bonferroni *post-hoc* tests was used for multiple comparisons. As behavioral experiments consisted of groups with unequal sample sizes, Bonferroni *post-hoc* tests were used due to the effect of unequal sample sizes on Tukey’s *post-hoc* method (Smith, 1971; Shingala and Rajyaguru, 2015). For calcium imaging experiments, paired *t*-tests were used for the comparison of calcium transients data sets before and during drug application, which had Gaussian distribution and similar variance as indicated. Repeated measures one-way ANOVA with Dunnett *post-hoc* tests were used for time course analysis of calcium transients compared to predrug ACSF vehicle administration at 10 min, as the Dunnett *post-hoc* method is used to test multiple experimental groups against a single control group (Dunnett, 1955; McHugh, 2011).

## Results

Previous anatomical data have demonstrated that CGRP is an important molecular marker of the CeLCand the parabrachial nucleus is the exclusive source of CGRP to the CeA (Schwaber et al., 1988; Honkaniemi et al., 1990; Dobolyi et al., 2005; Lu et al., 2015; Chen et al., 2018; Huang et al., 2021). We have previously shown that CGRP in the amygdala can exacerbate nocifensive and behavioral responses in normal rats (Han et al., 2010), and selective CGRP1 receptor antagonists can reverse pain-related plasticity in male rats in an arthritis pain model (Han et al., 2005). In this study, we investigated the role of CGRP in neuropathic pain-related amygdala function and behaviors in both sexes. We tested the hypothesis that the CGRP1 receptor is involved in neuropathic pain-related amygdala activity, and that blockade of this receptor in the amygdala could inhibit neuropathic pain behaviors in male and female rats.

### Expression Levels of CGRP and CGRP1 Receptor Components in the CeA in Neuropathic Pain

**Figure 1:**
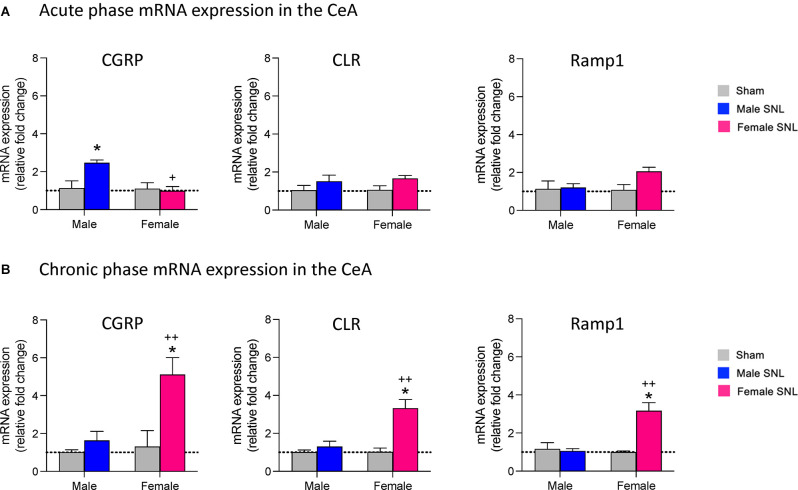
mRNA expression levels of CGRP and CGRP1 receptor components in the spinal nerve ligation (SNL) model of neuropathic pain. At both the acute (1 week) **(A)** and chronic (4 week) **(B)** phases of the SNL model, brains were extracted and the CeA dissected out for mRNA analysis. qRT-PCR analysis of mRNA expression levels for CGRP and two CGRP1 receptor components (calcitonin receptor-like receptor, CLR; receptor activity-modifying protein 1, Ramp1) was performed on CeA tissue at both time points. Data were normalized to the male sham group in both the acute and chronic phases. **(A)** CGRP mRNA expression in the acute phase was upregulated only in male SNL rats (*n* = 6). Female SNL rats (*n* = 6) had significantly lower CGRP expression than male SNL rats. No significant changes of CGRP1 receptor component expression were found. **(B)** CGRP mRNA expression in the chronic phase was significantly upregulated in female (*n* = 6) but not male (*n* = 6) SNL rats. CLR and Ramp1 receptor components were also upregulated only in female SNL rats. Gene expression fold change was calculated using the delta-delta Ct method, geometric mean of β-actin, Rpl3, and Rpl29 used as internal marker. Two-way ANOVA with Tukey *post-hoc* tests was used as sample sizes were equal for each group (see Section “Statistical Analysis”). Bar histograms show mean ± SEM. *P < 0.05, compared to male sham; ^+^, ^++^ *P* < 0.05, 0.01 compared to male SNL. See “Results” (“Expression Levels of CGRP and CGRP1 Receptor Components in the CeA in Neuropathic Pain”) Section for details of the statistical analysis.

We first examined mRNA expression levels of CGRP in the right CeA at the 1-week acute stage and 4-week chronic stage of the SNL model of neuropathic pain (see Section “Neuropathic Pain Model”). We found that SNL significantly increased CGRP mRNA expression in the CeA of male rats (*n* = 6) compared to sham (*n* = 6) at the acute phase (*P* < 0.05, *F*_3,20_ = 5.608, two-way ANOVA with Tukey *post-hoc* tests; Figure 1A), but no significant differences in CGRP expression levels were seen between female SNL (*n* = 6) and female sham (*n* = 6) rats at this stage. In fact, female SNL rats had significantly lower CGRP expression than male SNL rats at the acute phase (*P* < 0.05, *F*_3,20_ = 7.127, two-way ANOVA with Tukey *post-hoc* tests; Figure 1A). We also examined mRNA expression levels in the CeA of two CGRP1 receptor components, CLR and Ramp1 (McLatchie et al., 1998; Dickerson, 2013; Edvinsson and Warfvinge, 2013; Edvinsson et al., 2020). No significant differences in CLR or Ramp1 expression were found in the SNL model at the acute phase for males or females.

Interestingly, the opposite pattern of CGRP mRNA expression was seen at the chronic 4-week stage of neuropathic pain. Female SNL rats (*n* = 6) had significantly upregulated CGRP expression in the CeA compared to sham (*n* = 6; *P* < 0.05, *F*_3,20_ = 9.053, two-way ANOVA with Tukey *post-hoc* tests; Figure 1B), while there were no significant differences in CGRP expression levels between male SNL (*n* = 6) and male sham (*n* = 6) rats. CGRP mRNA expression was significantly higher in female SNL rats than male SNL rats at the chronic phase (*P* < 0.01, *F*_3,20_ = 6.779, two-way ANOVA with Tukey *post-hoc* tests; Figure 1B). For both CGRP receptor components, female SNL rats also had significantly upregulated mRNA expression in the CeA compared to that of female sham rats (CLR: *P* < 0.05, *F*_3,20_ = 11.24, two-way ANOVA with Tukey *post-hoc* tests; Ramp1: *P* < 0.05, *F*_3,20_ = 11.59, two-way ANOVA with Tukey *post-hoc* tests; Figure 1B) as well as significantly higher expression compared to male SNL rats (CLR: *P* < 0.01, *F*_3,20_ = 7.259, two-way ANOVA with Tukey *post-hoc* tests; Ramp1: *P* < 0.01, *F*_3,20_ = 7.681; Figure 1B). There were no significant differences in expression levels of either CLR or Ramp1 between male SNL and male sham rats during the chronic phase.

### Effects of CGRP1 Receptor Blockade in the CeA on Chronic Neuropathic Pain Behaviors

To investigate the role of CGRP in the CeA in chronic neuropathic pain-related behavior, we administered a selective CGRP1 receptor antagonist (CGRP 8-37, 100 μM in microdialysis probe) into the CeA of male and female rats 4 weeks after SNL surgery (see Section “Drug Application in Awake Animals”). The concentration of CGRP 8-37 in the microdialysis probe was 100-fold higher than the intended target concentration in the brain tissue to account for the concentration gradient across the dialysis membrane and diffusion in the brain tissue (Mazzitelli and Neugebauer, 2019; Hein et al., 2021).

**Figure 2:**
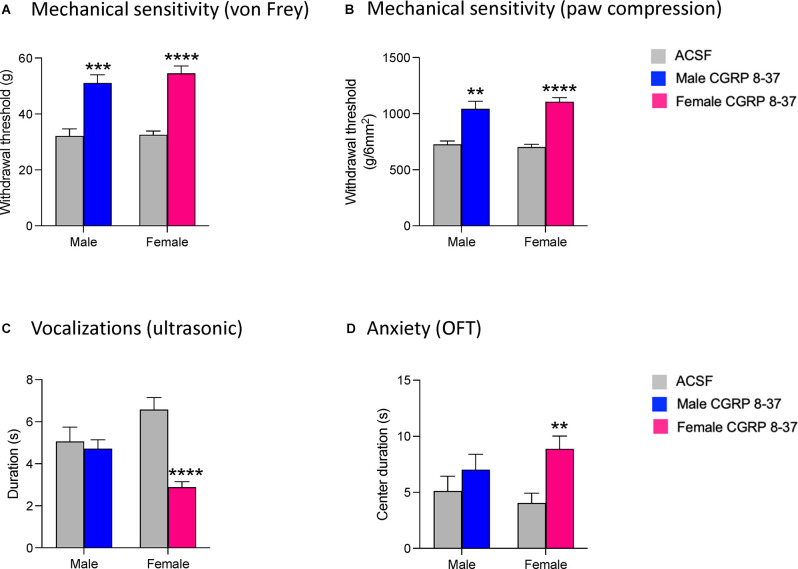
Effects of CGRP1 receptor blockade in the CeA on chronic neuropathic pain behaviors. Pain-related behavioral assays were performed 4 weeks after SNL surgery. Mechanical withdrawal thresholds were measured by electronic von Frey **(A)** and paw compression **(B)** tests. CGRP 8-37 (100 μM in microdialysis probe) administration into the CeA significantly increased withdrawal thresholds in both male (*n* = 12) and female (*n* = 24) SNL rats. **, ***, ****P < 0.01, 0.001, 0.0001, repeated measures ANOVA with Bonferroni *post-hoc* tests, compared to predrug ACSF vehicle. **(C)** Duration of ultrasonic vocalizations evoked by brief (10 s) noxious (500 g /6 mm^2^) mechanical compression of the affected hindpaw. CGRP 8-37 administration into CeA significantly decreased ultrasonic vocalizations in female (*n* = 24) but not male (*n* = 12) SNL rats compared to predrug ACSF vehicle. ****P < 0.0001, repeated measures ANOVA with Bonferroni *post-hoc* tests. **(D)** CGRP 8-37 administration into CeA significantly decreased anxiety-like behaviors in female (*n* = 24) but not male (*n* = 12) SNL rats as measured by increased duration (s) of time spent in the center of the open field test (OFT). **P < 0.01, ANOVA with Bonferroni *post-hoc* tests. Bonferroni *post-hoc* tests were used as sample sizes were unequal for each group (see Section “Statistical Analysis”). Bar histograms show mean ± SEM.

We first examined mechanical sensitivity using an electronic von Frey anesthesiometer (see Section “Mechanosensitivity”). CGRP 8-37 administration significantly increased the hindlimb withdrawal thresholds in both male (*n* = 12, *P* < 0.001) and female (*n* = 24, *P* < 0.0001) SNL rats compared to ACSF vehicle (see Figure 2A). There was a significant effect of CGRP1 receptor blockade (*P* < 0.0001, *F*_1,29_ = 58.56) but not sex (*P* = 0.5587, *F*_1,29_ = 0.3500) in the Von Frey test. Similarly, CGRP 8-37 administration into the CeA significantly decreased mechanical sensitivity in the paw compression test (reflex response evoked by gradually increasing the intensity of left hindpaw compression with a calibrated forceps, see Section “Mechanosensitivity”) in both male (*P* < 0.01) and female (*P* < 0.0001) SNL rats compared to ACSF vehicle (see Figure 2B). There was a significant effect of CGRP1 receptor blockade (*P* < 0.0001, *F*_1,29_ = 49.63) but not sex (*P* = 0.6238, *F*_1,29_ = 0.2459) on withdrawal thresholds using the paw compression test. For the statistical analyses of mechanical withdrawal thresholds in both the von Frey and paw compression tests, repeated measures two-way ANOVA with Bonferroni *post-hoc* tests was used (see Section “Statistical Analysis”).

We then examined emotional responses by measuring ultrasonic vocalizations in response to a noxious stimulus on the left hindpaw (see Section “Emotional-Affective Responses”). CGRP 8-37 administration significantly decreased the total duration of ultrasonic vocalizations in female SNL rats (*n* = 24, *P* < 0.0001, *F*_3, 29_ = 8.226; see Figure 2C), but not in male SNL rats (*n* = 12) compared to ACSF vehicle. For the statistical analysis of ultrasonic vocalization durations in males and females, repeated-measures two-way ANOVA with Bonferroni *post-hoc* tests was used (see Section “Statistical Analysis”).

Finally, we examined anxiety-like behaviors by measuring the time spent in the center zone of the OFT (see Section “Anxiety-Like Behavior”). Female SNL rats that received CGRP 8-37 in the CeA (*n* = 24) spent significantly more time in the center of the OFT compared to female SNL rats that received ACSF vehicle (*n* = 24, *P* < 0.01, *F*_3,65_ = 6.369; Figure 2D). In contrast, CGRP 8-37 administration into CeA had no significant effect in male SNL rats (*n* = 12) compared to ACSF vehicle (*n* = 12). Importantly, no significant differences in locomotor activity were observed between the ACSF-treated group and the CGRP 8-37-treated group (male, *P* > 0.9999; female, *P* = 0.9878), indicating that the differences in anxiety-like behavior were not due to an increase in spontaneous activity. For the statistical analysis of center duration in the OFT for males and females, two-way ANOVA with Bonferroni *post-hoc* tests was used (see Section “Statistical Analysis”).

### Effects of CGRP1 Receptor Blockade in the CeA on CeA-CRF Neuronal Activity in Chronic Neuropathic Pain

**Figure 3:**
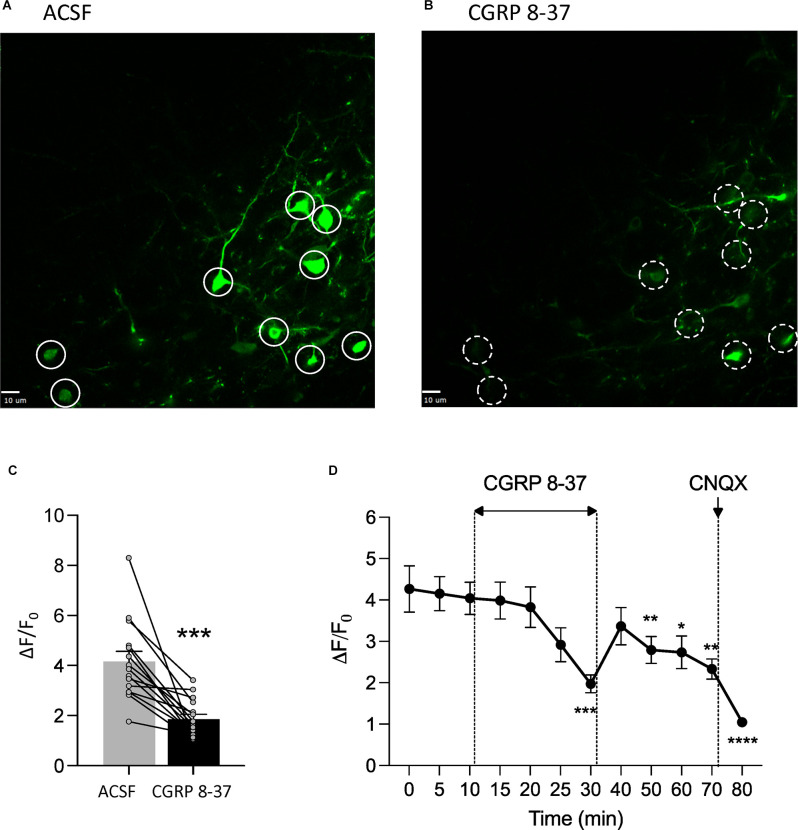
CGRP1 receptor blockade inhibits calcium transients in CeA-CRF neurons in chronic neuropathic pain. *In vitro* calcium imaging of CeA-CRF neurons in brain slices from chronic SNL rats was performed using a multiphoton imaging system (see Section “Calcium Imaging”). **(A,B)** Images show calcium transients in CeA-CRF neurons expressing a fluorescent calcium indicator (GCaMP7s) in response to electrical stimulation (1.5 mA, 0.6 ms) of presumed parabrachial afferents. Solid white circles **(A)** indicate neurons showing synaptically evoked fluorescence in the presence of ACSF vehicle; dashed white circles **(B)** indicate the same neurons after application of CGRP 8-37 (1 μM, 20 min). Scale bars represent 10 μM. **(C)** CGRP 8-37 decreased calcium transients in CeA-CRF neurons (*n* = 18 neurons from four rats) expressing GCaMP7s. ***P < 0.001, paired t-test, compared to predrug ACSF vehicle. **(D)** Time course analysis of calcium signals from CeA-CRF neurons during application of ACSF, CGRP 8-37, washout in ACSF, and CNQX (20 μM). *, **, ***, ****P < 0.05, 0.01, 0.001, 0.0001, repeated measures ANOVA with Dunnett *post-hoc* tests, compared to predrug ACSF vehicle at 10 min (see Section “Statistical Analysis”). Bar histograms show mean ± SEM.

To determine the neuronal effects of CGRP 8-37 in the amygdala as a potential basis of the inhibitory behavioral effects described in Section “Effects of CGRP1 Receptor Blockade in the CeA on Chronic Neuropathic Pain Behaviors”, we measured synaptically-evoked calcium signals in CeA-CRF neurons, which receive CGRP input from the parabrachial area and can modulate pain (Neugebauer et al., 2020; Mazzitelli et al., 2021, 2022), before and after drug application (Figure 3). Calcium transients were evoked in CRF neurons expressing a fluorescent calcium indicator (GCaMP7s) by electrical stimulation (1.5 mA, 0.6 ms) of presumed parabrachial afferents (see Section “Calcium Imaging”). Evoking CeA neuronal activity through the stimulation of parabrachial afferents has been a well-documented technique in previous studies from our group (Neugebauer et al., 2003; Fu and Neugebauer, 2008; Han et al., 2010; Ren et al., 2013; Thompson et al., 2018; Hein et al., 2021) and others (Ikeda et al., 2007; Watabe et al., 2013; Miyazawa et al., 2018; Yamamoto et al., 2021). The organization of parabrachial afferents to the amygdala has been well-characterized, with parabrachial fiber tracts shown to run dorsomedial to the CeA and ventral to but outside of the caudate-putamen area (Sarhan et al., 2005). The parabrachial afferent pathway to the CeA can be activated either by electrical (Neugebauer et al., 2003; Ikeda et al., 2007; Hein et al., 2021) or optogenetic (Sugimura et al., 2016; Hein et al., 2021) stimulation. Here neurons were visualized and signals were recorded following electrical stimulation using a multiphoton imaging system. Neurons that showed an increase in calcium signals in response to electrical stimulation were selected for further analysis. CGRP 8-37 (1 μM, 20 min) significantly decreased calcium transients in CeA-CRF neurons (*n* = 18 neurons from four rats; *P* < 0.001, paired t-test, compared to predrug ACSF vehicle; Figure 3C). Time course analysis revealed a significant effect of CGRP 8-37 treatment on calcium signals from CeA-CRF neurons (*P* < 0.0001, *F*_15,150_ = 15.60, one-way ANOVA with repeated measures and Dunnett *post-hoc* test; see Section “Statistical Analysis”; Figure 3D).

## Discussion

This study explored the role of CGRP in sensory and affective pain-related behaviors as well as in pain-related amygdala function in male and female rats in a neuropathic pain model. We previously showed that selective CGRP1 receptor antagonists (CGRP 8-37 and BIBN4096BS) can inhibit CeA neuronal excitability *in vitro* and can reduce withdrawal reflexes and vocalizations in awake male animals in an acute arthritis pain model (Han et al., 2005). However, it is unclear if these findings extend to a chronic pain condition and whether this inhibition elicits similar behavioral responses in the female sex. The key novelties in this study are the sex-specific characterizations of CGRP and CGRP1 receptor component expression levels in the CeA at both acute and chronic stages of neuropathic pain, the differential behavioral modulation by CGRP1 signaling in the CeA between males and females at the chronic stage, and the effects of CGRP1 receptor blockade on CeA-CRF neurons in chronic neuropathic pain.

Abundant preclinical (Neugebauer et al., 2009; Veinante et al., 2013; Neugebauer, 2015, 2020; Thompson and Neugebauer, 2017; Allen et al., 2021) and clinical (Baliki et al., 2006, 2008; Geha et al., 2007; Kulkarni et al., 2007; Liu et al., 2010; Vachon-Presseau et al., 2012, 2016; Simons et al., 2014) evidence has linked the amygdala to the emotional-affective aspects of pain and pain modulation. The lateral and capsular regions of the CeAconstitute the “nociceptive amygdala” due to the high content of neurons encoding nociceptive information and modulating pain-related behaviors (Neugebauer et al., 2004, 2020; Neugebauer, 2015). Changes in the activity of these neurons have been demonstrated inacute inflammatory pain (Adedoyin et al., 2010; Sugimura et al., 2016; Shinohara et al., 2017; Miyazawa et al., 2018), in colitis (Han and Neugebauer, 2004), in muscle pain (Cheng et al., 2011), in neuropathic pain (Ikeda et al., 2007; Gonçalves and Dickenson, 2012; Nakao et al., 2012; Ji et al., 2017), and in arthritis pain models (Neugebauer and Li, 2003; Bird et al., 2005; Han et al., 2005; Li and Neugebauer, 2006; Ji and Neugebauer, 2007, 2009; Fu and Neugebauer, 2008; Fu et al., 2008; Ji et al., 2009; Ren and Neugebauer, 2010; Ren et al., 2011, 2013). Decreasing amygdala activity has been shown to inhibit pain-related behavior in arthritic (Han and Neugebauer, 2005; Ji et al., 2007, 2010; Fu and Neugebauer, 2008; Palazzo et al., 2008; Ji and Neugebauer, 2009; Grégoire and Neugebauer, 2013; Ren et al., 2013; Medina et al., 2014; Thompson et al., 2015; Kim et al., 2017; Mazzitelli and Neugebauer, 2019; Mazzitelli et al., 2021), acute inflammatory (Kolber et al., 2010; Palazzo et al., 2011; Sugimoto et al., 2021), visceral (Crock et al., 2012), widespread nociplastic (Yajima et al., 2022), and neuropathic (Pedersen et al., 2007; Ansah et al., 2010; Jiang et al., 2014; Ji et al., 2017; Seno et al., 2018; Wilson et al., 2019; Mazzitelli et al., 2022) pain models.

The lateral and capsular divisions of the CeA are the targets of nociceptive input from the spino-parabrachio-amygdaloid pain pathway (Gauriau and Bernard, 2002), where neurons from the lateral pontine parabrachial area provide the main if not exclusive source of CGRP within the amygdale (Dobolyi et al., 2005; D’Hanis et al., 2007; Shinohara et al., 2017; Palmiter, 2018). CGRP has previously been suggested to act as an important modulator of synaptic plasticity within the amygdala, as its exogenous application onto brain slices from normal rats increased both excitatory transmission at the parabrachio-amygdaloid synapse as well as excitability of neurons in the lateral and capsular CeA through N-methyl-D-aspartate (NMDA)- and PKA-dependent mechanisms (Han et al., 2010). Furthermore, the stereotaxic administration of CGRP into the right CeA of awake rats increased emotional responses (audible and ultrasonic vocalizations) to noxious stimuli and induced mechanical hypersensitivity (lowered hindlimb withdrawal thresholds; Han et al., 2010), and CGRP1 receptor blockade in the CeA reduced mechanical hypersensitivity and emotional responses in an acute arthritis pain model (Han et al., 2005). Similarly, mechanical sensitivity in the formalin test was significantly decreased 6 h post-inflammation in CGRP knockout mice, while acute nociceptive behavior was reduced only at 20–25 min after injection (Shinohara et al., 2017). The role of CGRP is therefore a critical link to investigate in the mechanistic analysis of pain-related processing in the amygdala.

A substantial portion (35%–42%) of CRF neurons, which serve output functions in the lateral and capsular regions of the CeA, receive input from CGRP-containing parabrachial afferents (Kruger et al., 1988; Shimada et al., 1989; Harrigan et al., 1994; Dobolyi et al., 2005; D’Hanis et al., 2007; Chen et al., 2018; Palmiter, 2018). As such, CGRP may strengthen the synaptic drive onto CRF neurons and influence amygdala output activity (Han et al., 2010; Okutsu et al., 2017; Shinohara et al., 2017). Here our multiphoton calcium imaging experiments revealed that CGRP1 receptor blockade in a chronic neuropathic pain model decreased CeA-CRF neuronal activity, which indicates endogenous CGRP release and would translate into decreased output from these neurons. As CRF neurons project to numerous extra-amygdalar targets involved in the modulation of averse-affective pain behaviors (Beckerman et al., 2013; Pomrenze et al., 2015, 2019; Dedic et al., 2018; de Guglielmo et al., 2019; Neugebauer et al., 2020), we expect that the deactivation of CeA-CRF neurons would correspond with beneficial behavioral effects observed here in a neuropathic pain model. This is consistent with our previous studies showing that optogenetic silencing of CeA-CRF neurons inhibits pain behaviors (Mazzitelli et al., 2021, 2022). Our data implicate CGRP1 receptors in the pain facilitating role of CRF neurons. The calcium imaging data complement data from our behavioral experiments to suggest that reduced CeA-CRF neuronal activity plays a critical contribution to the facilitatory behavioral responses that we saw from CGRP1 receptor antagonism in chronic neuropathic pain. However, the full downstream consequences of reduced CeA-CRF neuronal output remain to be determined.

Information about sex-specific roles for CGRP in pain modulation is rather limited, though a few studies have focused on sex-differential expression levels in the spinal cord. In the spinal trigeminal nucleus caudalis (SpVc), naïve male rats showed higher baseline mRNA levels of CLR, Ramp1, and the third receptor component, RCP, but not of CGRP when compared to female counterparts (Stucky et al., 2011). Surprisingly, expression of the CGRP-related genes increased in both sexes 30 min after dural application of either an inflammatory cocktail or a vehicle control, though this upregulation was larger in females than in males. As both the inflammatory and control treatments promoted increased CGRP-related gene expression, the authors attributed this upregulation to mechanical stimulation from cannula implantation as opposed to meningeal inflammation (Stucky et al., 2011). However, a later study found SpVc protein levels of RCP but not CLR were higher in naïve females than in males (Ji et al., 2019). In the periphery, naïve female rats were shown to have fewer CGRP-immunoreactive dorsal root ganglion (DRG) neurons than their male counterparts, though ovariectomy produced a significant increase in immunoreactive neurons (Yang et al., 1998). A recent study reported no significant sex differences in CGRP-immunostaining cells in the DRG 7 days after the induction of neuropathic pain using the spared nerve injury (SNI) model (Ahlström et al., 2021). To our knowledge, this study is the first to report neuropathic pain stage-specific sex differences in mRNA expression levels of CGRP and its receptor components within the brain. Additional investigation is needed to confirm sex differences in CGRP and CGRP receptor expression at the protein level and characterize pain-related transcriptional and translational regulatory mechanisms of CGRP and its receptor components at each level of the neuraxis in male and female animals. As a note of caution, we did not determine or analyze separately the different stages of the estrous cycle because previous evidence because our previous work (Chen et al., 2020) and the current study found that sexually dimorphic effects were robust, readily detectable, and statistically significant, even though we did not control for the estrous stage of females, suggesting that the estrous cycle may not be a major factor in the outcomes of our studies. Actually, the value of testing female rodents at different stages of the estrous cycle is somewhat debatable (Greenspan et al., 2007).

Potential sex differences in CGRP-related pain behaviors have also been explored. Within the brain, a migraine model of dural CGRP administration produced hypersensitivity responses in female but not male rats (Avona et al., 2019). In the SpVc, knockdown of RCP expression with short hairpin RNA (shRNA) attenuated mechanical facial allodynia produced by noxious chemical stimulation of the meninges in both male and female rats (Ji et al., 2019). However, another group found that intrathecal injection of CGRP receptor antagonists (CGRP 8-37 orolcegepant) attenuated mechanical hypersensitivity in a female-specific manner in both a hyperalgesic priming model and at the chronic phase of the SNI neuropathic pain model (Paige et al., 2022). In the same study, intrathecal injection of CGRP caused prolonged mechanical hypersensitivity only in female mice, an effect that was blocked by systemic pretreatment with olcegepant (Paige et al., 2022). These data are consistent with our current finding that CGRP receptor antagonism reduces mechanical hypersensitivity in a neuropathic pain model to a greater extent in female animals. Additionally, since we found that this treatment strategy alleviated other dimensions of pain (emotional-affective responses and anxiety-like behaviors) more strongly in female rats, our collective data provide support for the utilization of CGRP receptor-blocking therapies in the central nervous system for female chronic neuropathic pain patients.

Increasing evidence across the pain field has revealed sexually dimorphic mechanisms that contribute to pain development and maintenance (recently reviewed in Presto et al., 2022). Studies from the periphery and spinal cord have illustrated a male-predominant reliance on macrophage-related (Liu et al., 2020; Rudjito et al., 2021) and microglia-related (Sorge et al., 2011, 2015; Taves et al., 2016; Agalave et al., 2021) pain processing mechanisms. However, the underlying pain modulatory processes for females are complex and not well-characterized. Particularly within the brain, sex-specific signaling mechanisms are largely unknown. Our data may be the first to show sex differences in pain-related behavior following CGRP receptor blockade within the brain, which may be attributed to sex-specific molecular expression profiles throughout neuropathic pain development. Though CGRP expression in the CeA was not upregulated in male rats at the chronic stage, blockade of the CGRP1 receptor at this phase still showed inhibitory effects on sensory pain responses. This may reflect a more tonic role of CGRP signaling in males during the chronic phase, as CGRP also exhibits several physiological properties within the brain (Abushik et al., 2017; Borkum, 2019; Tian et al., 2020). As pain-related CGRP signaling in the CeA of male rats appears to play a significant role during the induction phase but primarily sensory role at the chronic stage of neuropathic pain modulation, our study could provide support that other mechanisms, such as those involved in neuroimmune signaling, may be more relevant for brain-related pain processing in males. Importantly, the impact of CGRP-related pain mechanisms may differ across the pain time course. Our previous studies have highlighted the beneficial effects of CGRP1 receptor blockade in the CeA on pain-related behaviors in males in relatively acute (arthritis) pain conditions (Han et al., 2005, 2010). The role of CGRP in the arthritis model may be more comparable to the acute 1-week stage of the SNL model presented here, in which a stronger upregulation of CGRP mRNA expression was observed in male compared to female SNL rats. The current data suggest that while CGRP signaling may play a critical role in brain-related pain processing at an acute stage in males, other modulatory mechanisms may take on a more predominant role with regard to pain chronicity and maintenance. This illustrates the urgent need to investigate potential sexual dimorphisms in pain-related amygdala function across all stages of neuropathic pain development.

## Conclusion

The data may suggest that while CGRP-related signaling mechanisms play an important role in neuropathic-pain-related amygdala function, this influence likely differs with respect to the time course of pain development in males and females. CGRP1 receptor blockade in the amygdala may serve as a novel therapeutic strategy for chronic neuropathic pain relief, particularly among female chronic pain patients. Future investigation into the contributions of other pain modulatory mechanisms, such as the role of neuroimmune signaling in the transition from acute to chronic pain, is warranted in both sexes. Ultimately, our work provides support for the investigation of therapeutic targets, such as CGRP receptors, in both male and female subjects across different stages of neuropathic pain.

## Data Availability Statement

The original contributions presented in the study are included in the article, further inquiries can be directed to the corresponding author.

## Ethics Statement

The animal study was reviewed and approved by Institutional Animal Care and Use Committee (IACUC, protocol #14006) of Texas Tech University Health Sciences Center (TTUHSC).

## Author Contributions

PP: methodology, molecular, behavioral and imaging experiments, data analysis, writing original draft. VN: conceptualization, methodology, experimental design, supervision, editing and finalizing the manuscript, project administration, funding acquisition. All authors contributed to the article and approved the submitted version.

## Conflict of Interest

The authors declare that the research was conducted in the absence of any commercial or financial relationships that could be construed as a potential conflict of interest.

## Publisher’s Note

All claims expressed in this article are solely those of the authors and do not necessarily represent those of their affiliated organizations, or those of the publisher, the editors and the reviewers. Any product that may be evaluated in this article, or claim that may be made by its manufacturer, is not guaranteed or endorsed by the publisher.
